# BCG scar, socioeconomic and nutritional status: a study of newborns in urban area of Makassar, Indonesia

**DOI:** 10.1111/tmi.13232

**Published:** 2019-04-05

**Authors:** Aldian Irma Amaruddin, Sitti Wahyuni, Firdaus Hamid, Maisuri T. Chalid, Maria Yazdanbakhsh, Erliyani Sartono

**Affiliations:** ^1^ Department of Parasitology Faculty of Medicine Hasanuddin University Makassar Indonesia; ^2^ Department of Parasitology Leiden University Medical Center Leiden The Netherlands; ^3^ Department of Microbiology Faculty of Medicine Hasanuddin University Makassar Indonesia; ^4^ Department of Obstetrics and Gynaecology Faculty of Medicine Hasanuddin University Makassar Indonesia

**Keywords:** Bacille Calmette–Guérin scar, socioeconomic status, leptin, newborns, cicatrice du BCG, statut socioéconomique, leptine, nouveau‐nés

## Abstract

**Objective:**

To investigate factors that determine the response to Bacille Calmette–Guérin (BCG) vaccination in urban environments with respect to socioeconomic status (SES), prenatal exposure to infections or newborn's nutritional status.

**Methods:**

The study was conducted in an urban area, in Makassar, Indonesia. At baseline, 100 mother and newborns pair from high and low SES communities were included. Intestinal protozoa, soil transmitted helminths, total IgE, anti‐Hepatitis A Virus IgG and anti‐*Toxoplasma* IgG were measured to determine exposure to infections. Information on gestational age, birth weight/height and delivery status were collected. Weight‐for‐length *z*‐score, a proxy for newborns adiposity, was calculated. Leptin and adiponectin from cord sera were also measured. At 10 months of age, BCG scar size was measured from 59 infants. Statistical modelling was performed using multiple linear regression.

**Results:**

Both SES and birth nutritional status shape the response towards BCG vaccination at 10 months of age. Infants born to low SES families have smaller BCG scar size compared to infants born from high SES families and total IgE contributed to the reduced scar size. On the other hand, infants born with better nutritional status were found to have bigger BCG scar size but this association was abolished by leptin levels at birth.

**Conclusion:**

This study provides new insights into the importance of SES and leptin levels at birth on the development of BCG scar in 10 months old infants.

## Abstract (fr)

**Objectif:**

Investiguer les facteurs qui déterminent la réponse à la vaccination par le BCG en milieu urbain en ce qui concerne le statut socioéconomique (SSE), l'exposition prénatale aux infections ou l’état nutritionnel du nouveau‐né.

**Méthodes:**

L’étude a été menée dans une zone urbaine, à Makassar, en Indonésie. Au départ, 100 paires mère‐nouveau‐né issues de communautés à statut social élevé et faible ont été incluses. Les protozoaires intestinaux, les helminthes transmis par le sol, les IgE totales, les IgG anti‐virus de l'hépatite A et anti‐ *Toxoplasma* ont été mesurés pour déterminer l'exposition aux infections. Des informations sur l’âge gestationnel, le poids/taille à la naissance et l’état d'accouchement ont été collectées. Le z‐score poids‐pour la taille, un indicateur indirect de l'adiposité du nouveau‐né a été calculé. La leptine et l'adiponectine provenant de sérum des cordons ont également été mesurées. A l’âge de 10 mois, la taille des cicatrices de BCG a été mesurée chez 59 nourrissons. La modélisation statistique a été réalisée à l'aide d'une régression linéaire multiple.

**Résultats:**

Le statut socioéconomique et l’état nutritionnel à la naissance déterminent la réponse à la vaccination par le BCG à l’âge de 10 mois. La taille des cicatrices de BCG est plus petite chez les nourrissons nés de familles à statut socioéconomique faible comparée à celles chez ceux de familles à statut socioéconomique élevé et les IgE totales ont contribué à la réduction de la taille de ces cicatrices. En revanche, les bébés nés avec un meilleur état nutritionnel avaient une taille de cicatrice du BCG plus grande, mais cette association était supprimée par les niveaux de leptine à la naissance.

**Conclusion:**

Cette étude fournit de nouvelles informations sur l'importance du SSE et des niveaux de leptine à la naissance sur le développement d'une cicatrice du BCG chez des nourrissons âgés de 10 mois.

## Introduction

Tuberculosis (TB) is known as one of the top 10 diseases causing high mortality worldwide. In 2017, with 391 new cases per 100 000 population, Indonesia was among the top three countries with absolute numbers of incident TB cases 1.

Bacille Calmette–Guérin (BCG) is a live‐attenuated *Mycobacterium bovis* vaccine. It is the only available vaccine used to protect against TB disease, in particular meningitis and disseminated TB in children 2. BCG is one of the most widely used vaccines worldwide. In Indonesia, BCG vaccination is included in the Indonesian national immunisation programme and it is given to newborns at the age of 4–6 weeks. Beside its protective effects against TB, BCG vaccination has also shown to result in non‐specific lower mortality and morbidity during childhood 3, 4, 5. BCG vaccination induces a memory T‐helper‐1 (Th‐1) type response irrespective of when in life it is given 6. Studies have shown that reactions at the site of the BCG vaccination are associated with the production of Interferon gamma in response to the mycobacterial antigens. BCG scarification has been mentioned as a marker to a better survival and stronger immune response among BCG‐vaccinated children living in countries with higher mortality rates 7, 8.

Immune responses to vaccines are associated with multiple factors such as economic status, parasite infestation and nutrition. Nutritional status at birth reflects newborns adiposity and this might affect BCG vaccine response in these babies 9, 10. Adipocytes influence not only the endocrine system but also the immune response through several cytokine‐like mediators known as adipokines, which include leptin and adiponectin 11, 12. Adiponectin and leptin are considered the most important hormones related to adipose depots in modulating metabolism and energy homeostasis. It is thought that leptin can directly link nutritional status and pro‐inflammatory Th‐1 immune responses, while adiponectin possesses anti‐inflammatory properties 13, 14, 15, 16.

With respect to economic status, a study on rotavirus vaccine showed that the efficacy of this vaccine was lower in low‐ compared to high‐income countries 17. Moreover, within low income countries, a gradient in reduced efficacy was shown with decreasing Gross Domestic Products (GDP) 18. Low socioeconomic status (SES) has been linked to lack of adequate water, sanitation and poor hygiene practices 19 and higher exposure to infections 19, 20, 21, 22, such as intestinal parasites 23, 24, toxoplasma 25, 26 and hepatitis A 21, 27.

All these environmental aspects can have an impact on the immune system as shown by a study of twins in the USA where it was discovered that not only genetic but very importantly environmental factors can affect the immune system 28. This is confirmed by several studies involving low–middle‐income countries where geographical differences in immune profiles have been examined 29, 30, 31, 32.

Indonesia with its rapid economic development bears a large diversity in population life style and SES. This study is aimed to better understand factors that determine the response to BCG vaccination. Of particular interest is the nutritional status of the newborn and prenatal exposure to intestinal parasites, toxoplasma and hepatitis A infection. To this end, a study was conducted in three hospitals that serving high and low SES population. Maternal demographic data, socioeconomic characteristic, and infection status as well as their newborns nutritional status, total IgE (tIgE) and leptin levels were used for performing pathway analysis in order to better understand how BCG vaccine responses are shaped.

## Methods

### Ethics statement

The study was approved by the Health Research Ethical Committee, Faculty of Medicine, Hasanuddin University (ref.: 0685/H4.8.4.5.31/PP36‐KOMETIK/2014). This study was executed based on codes stated in Declaration of Helsinki and International Ethical Guidelines for Epidemiological Studies. Written informed consent was obtained from mothers for the collection of their samples and their newborn samples.

### Study population

The study population consisted of pregnant mothers and their born infants living in urban city of Makassar between January 2015 and May 2016. Pregnant women in the last trimester were recruited in government hospitals and private hospitals. Questionnaires were used to gather information regarding demographic, socioeconomic status and education. Based on Makassar minimum city wages, family income of IDR 60 million/year is used to define high (income equal/more than IDR 60 million/year) and low (income less than IDR 60 million/year) SES. Maternal characteristics such as gravidity, parity, miscarriage history or other medical conditions were also collected. Information about gestational age was estimated from last menstrual date.

Child information such as sex and delivery status were collected via the midwives or obstetricians through a questionnaire. Only infants born full term, delivered vaginally and healthy were included in the study. Birth weight and height were assessed of newborn wearing the minimum clothing, using baby weighing scale (GEA, Megapratama Medikalindo, Indonesia) and newborn length board. The weighing scale was calibrated using standardised weight as part of routine care. Weight‐for‐length *z*‐score (Wflz) at birth, a proxy of newborn adiposity and nutritional status was calculated according to the WHO references value 33, 34.

### BCG vaccination and scar measurement

Indonesia's vaccination programme requires every infant to be vaccinated with BCG at 6 weeks of age by staff from local primary health care centre. The vaccine given contains live‐attenuated *Mycobacterium bovis* Paris 1173‐P2 strain (Biofarma, Bandung, Indonesia). The resulting BCG scar was measured at 10 months of age by the same person from the research team. Mean diameter of scar size was calculated from diameters perpendicular to each other. Vaccination information which proved that the newborns were BCG‐vaccinated were copied from their health book or health card.

### Blood and stool sample collection

Maternal blood samples were collected in the last trimester of pregnancy while cord blood samples were collected right after delivery. All blood samples were transported to the laboratory at Hasanuddin University Medical Research Center (HUM‐RC) and processed within 4 h of collection. Maternal stool samples were collected and stored at −80 °C until further analysis.

### Parasitological examination

DNA isolation from stool was performed as described elsewhere 35, 36. Two panels of multiplex real‐time PCR were used to detect and quantify soil transmitted helminths and intestinal protozoa. Panel 1 targets hookworm (*Ancylostoma duodenale, Necator americanus*), *Ascaris lumbricoides*,* Trichuris trichuria* and *Strongyloides stercoralis* 35, 36, 37, 38, while panel 2 targets *Entamoeba histolytica, Dientamoeba fragilis, Giardia lamblia* and *Cryptosporidium* spp. 38, 39, 40, 41.

### Measurements of tIgE, anti‐Hepatitis A Virus IgG, anti‐*Toxoplasma gondii* IgG, leptin and adiponectin

Total IgE levels in maternal and cord samples were measured using ELISA technique as described previously 35. For this assay, maternal and cord sera were diluted 50 and two times in PBS containing 0.05% Tween20 respectively (Tween‐20, Sigma‐Aldrich, St. Louis, MO, USA). The results were expressed in International Units per millilitre (IU/ml).

Anti‐Hepatitis A Virus IgG (anti‐HAV IgG) and anti‐*Toxoplasma gondii* IgG (anti‐Toxoplasma IgG) in cord samples were measured at Department of Microbiology in LUMC as part of routine diagnostic procedures. Samples were considered to be positive for anti‐HAV IgG if the signal‐to‐cut‐off (S/CO) ratio was ≥1.00, while for anti‐Toxoplasma IgG, the samples were considered to be positive if the titre was ≥8 IU/ml.

Leptin and adiponectin levels were quantified using Human Leptin Duo Set and Human Adiponectin Duo Set (R&D System, Abingdon, UK) at Department of Parasitology in LUMC, according to the manufacturer's guidelines 42. Maternal sera were diluted 40 and 4000 times, while cord sera were diluted 10 and 10 000 times for measurement of leptin and adiponectin respectively. The values were expressed in ng/ml and μg/ml for leptin and adiponectin respectively.

### Statistical analysis and conceptual framework

Descriptive data were presented in mean ± standard deviation for normally distributed data and median (interquartile range, IQR) for non‐normally distributed data. The correlation between two continuous data was done using Pearson correlation for normally distributed data or Spearman's rank correlation for non‐normally distributed data. Prevalence was calculated as percentage of collected data. Pearson chi‐square test was used to compare the prevalence of infection between two groups.

To obtain normally distributed data, maternal tIgE, cord leptin and adiponectin level were log10‐transformed. Student *t*‐test was then used to compare the mean differences between two groups. Since the levels of cord blood tIgE, anti‐HAV IgG and anti‐Toxoplasma IgG were not normally distributed even after transformation, the comparison between two groups was performed using Mann–Whitney *U* test.

**Figure 1:**
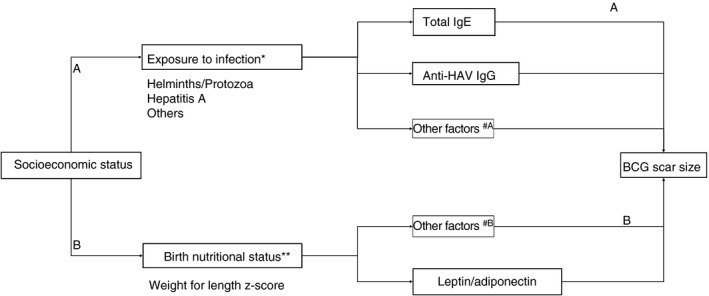
Conceptual framework of the study. The analysis of the association between Bacille Calmette–Guérin (BCG) scar size and SES through two different pathways. The analysis asked whether SES contributes to the outcome of BCG vaccination through (A) prenatal exposure to infection or (B) birth nutritional status pathway, using linear regression adjusted with gestational age and child gender for both pathways. *total IgE and anti‐Hepatitis A IgG were measured as proxy of current or previous exposure to infections. **weight‐for‐length *z*‐score was measured as a proxy of nutritional status at birth. ^#A/#B^ Other factors that were not assessed in pathway A or B.

The conceptual framework in Figure 1 was used to answer our research question. First, (A) we assessed whether the effect of SES to the size of BCG scar is through exposure to infections. Second, (B) we assessed the contribution of SES to the size of BCG scar is through the nutritional status pathway and whether leptin or adiponectin have a mediatory role in this path.

We primarily performed a linear regression to obtain estimate coefficients of SES on the size of BCG scar. Next, to test the contribution of SES on BCG scar through infection pathway (A), this model was adjusted for tIgE, anti‐HAV IgG or anti‐Toxoplasma IgG as a proxy of prenatal exposure to infection. In pathway (B), we first assessed association between SES and WfLz, a proxy of adiposity at birth. Subsequently, the effect of WfLz on BCG outcome was modelled by adjusting with leptin and adiponectin. Mediation was considered to occur if 1 all the crude association between variables tested in this pathway were significant; 2 the association disappeared and the effects attenuated when the model was adjusted for tIgE, leptin or adiponectin.

All models above were analysed using linear regression with adjustments for gestational age, sex and other variable that we found relevant to be account as confounder. Statistical test was considered significant at *P* < 0.05. All data were analysed using IBM Statistical Package for the Social Sciences (SPSS) Statistics version (IBM‐SPSS Inc., Chicago, IL, USA).

## Results

### Characteristics of the study population

Maternal characteristics in low and high SES are presented in Table 1. The prevalence of any intestinal protozoa was not different between low and high SES. Although no significant differences in the prevalence of soil transmitted helminths was seen between two groups, the prevalence of *A. lumbricoides* (12% *vs*. 4%) and *T. trichiura* (12% *vs*. 6%) was slightly higher in low than in high SES mothers respectively. The prevalence of hookworm (2%) and *S. stercoralis (2%)* was very low.

**Table 1:** Baseline characteristics of the study population of Low (low SES) and High (high SES) socioeconomic status

	Total		Low SES		High SES		*P* value^a^
	*N*	Results	*N*	Results	*N*	Results	
*Mothers*							
Age, years, mean ± SD	100	27.44 ± 4.90	50	26.71 ± 5.27	50	28.18 ± 4.42	0.135
Primigravida, *N*,* n* (%)	100	53 (53)	50	30 (60)	50	23 (46)	0.229
Intestinal parasites infection							
Any helminth, *N*,* n* (%)	100	16 (16)	50	9 (18)	50	7 (14)	0.585
*Ascaris lumbricoides*,* N*,* n* (%)	100	8 (8)	50	6 (12)	50	2 (4)	0.140
*Trichuris trichiura*,* N*,* n* (%)	100	9 (9)	50	6 (12)	50	3 (6)	0.295
Any protozoa, *N*,* n* (%)	100	20 (20)	50	17 (34)	50	13 (26)	0.517
*Entamoeba histolytica, N*,* n* (%*)*	100	3 (3)	50	2 (4)	50	1 (2)	0.558
*Dientamoeba fragilis, N*,* n* (%)	100	26 (26)	50	14 (28)	50	12 (24)	0.648
*Giardia lamblia, N*,* n* (%)	100	4 (4)	50	2 (4)	50	2 (4)	1.000
*Cryptosporidium*. spp *N*,* n* (%)	100	1 (2)	50	1 (2)	50	0 (0)	0.315
Any intestinal parasites, *N*,* n* (%)	100	42 (42)	50	23 (46)	50	19 (38)	0.418
Total IgE, IU/ml, geomean (95% CI)	100	180.82 (142.31–229.75)	50	248.08 (175.36–350.96)	50	131.80(95.94–181.06)	**0.008**
Leptin, ng/ml, geomean (95% CI)	100	30.28 (26.40–34.73)	50	29.45 (24.00–36.12)	50	31.15 (25.77–37.65)	0.687
Adiponectin, μg/ml, geomean (95% CI)	100	3.41 (3.00–3.87)	50	3.58(3.04–4.23)	50	3.25 (2.67–3.96)	0.453
*Newborns*							
Sex, female *N*,* n* (%)	100	50 (50)	50	28 (56)	50	22 (44)	0.317
First born, *N*,* n* (%)	100	52 (52)	50	30 (60)	50	22 (44)	0.161
Gestational age, weeks, mean ± SD	100	39.71 ± 1.6	50	39.69 ± 1.68	50	39.73 ± 1.53	0.902
Birth weight, grams, mean ± SD	100	3130.5 ± 402.76	50	3194.64 ± 368.26	50	3156.67 ± 415.79	0.666
Weight‐for‐length *z*‐ score at birth, mean ± SD	100	−0.50 ± 0.63	50	−0.47 ± 0.52	50	−0.46 ± 0.65	0.887
Total IgE, IU/ml, median (IQR)	100	0.07 (0.07–0.21)	50	0.12(0.07–0.76)	50	0.07(0.07–0.07)	**<0.001**
Ratio S/CO anti‐Hepatitis A Virus IgG, median (IQR)	98	9.60 (7.77–10.37)	49	9.93 (9.07–10.46)	49	9.33 (0.30–10.28)	0.088
Anti‐Hepatitis A Virus IgG seropositivity, *N*,* n* (%)	98	77 (78.6)	49	42 (85.7)	49	35 (71.4)	0.139
Anti‐*Toxoplasma* IgG titres, IU/ml, median (IQR)	100	0.00 (0.00–41.25)	50	0.00 (0.00–44.25)	50	0.00 (0.00–37.00)	0.846
Anti‐*Toxoplasma* IgG seropositivity, *N*,* n* (%)	100	35 (35)	50	20 (40)	50	15 (30)	0.402
Leptin, ng/ml, geomean (95% CI)	100	10.04 (8.30–12.14)	50	9.15 (7.17–11.68)	50	11.01 (8.17–14.84)	0.336
Adiponectin, μg/ml, geomean (95% CI)	100	16.44 (14.35–18.83)	50	17.73 (14.68–21.41)	50	15.24 (12.48–18.61)	0.271

Data presented as number of positives (n) of the total population (N) and as percentage of total population (%).

SD, standard deviation; IU, international unit; S/CO, signal‐to‐cut‐off.

a Unadjusted.

Bold: *P* value < 0.05.

The levels of tIgE were significantly higher in low than in high SES mothers [geomean (95% confidence interval, CI), 248.08 IU/ml (175.36–350.96 IU/ml) and 131.80 IU/ml (95.94–181.06 IU/ml) respectively; *P* = 0.008] (Table 1). Anti‐HAV IgG S/CO ratio was slightly higher in mother with low than in high SES women [median (IQR), 9.93 (9.07–10.46) and 9.33 (0.30–10.28) respectively; *P* = 0.088] (Table 1). There were no differences in the levels of IgG antibody against *T. gondii* between two groups. Similarly, adiponectin or leptin levels were not different between low and high SES mothers.

Of 100 newborns that were included in the study, no significant differences in sex ratio, nor gestational age between low and high SES group was seen. Birth weight, height and Wflz were not different between low and high SES newborns (Table 1). The level of tIgE in cord blood was significantly higher in low (median [IQR], 0.12 IU/ml [0.07–0.76]) compared to high SES (median [IQR], 0.07 IU/ml [0.07–0.07]) newborns (*P *<* *0.001) (Table 1).

The levels of leptin and adiponectin were not different between high and low SES newborns. When gender was considered, girls either form high or low socioeconomic status tended to have higher leptin levels than boys (High SES: geomean [95% CI] = 15.25 ng/ml [9.76–23.84] *vs*. 8.52 ng/ml [5.74–12.69], *P* = 0.103; Low SES: 10.90 [8.09–14.70] *vs*. 7.31 ng/ml [4.84–11.03], *P* = 0.051), in girls and boys respectively.

During follow‐up at 10 months of age, we were able to collect data from 59 of 100 infants (29 from low SES and 30 from high SES). There were no differences in sex ratio between these high and low SES groups. In terms of weight, high SES infants gained more weight than low SES infants (mean ± SD, 5103.33 ± 534.65 *vs*. 4777.58 ± 466.28; *P* = 0.016). The major reason of loss to follow‐up was families moving out of the city. There were no differences in gestational age, sex, birth weight, Wflz, tIgE, leptin nor adiponectin levels between those who remained in the study and those who were lost to follow‐up.

### The effect of SES and exposure to infection on the size of BCG scar

**Figure 2:**
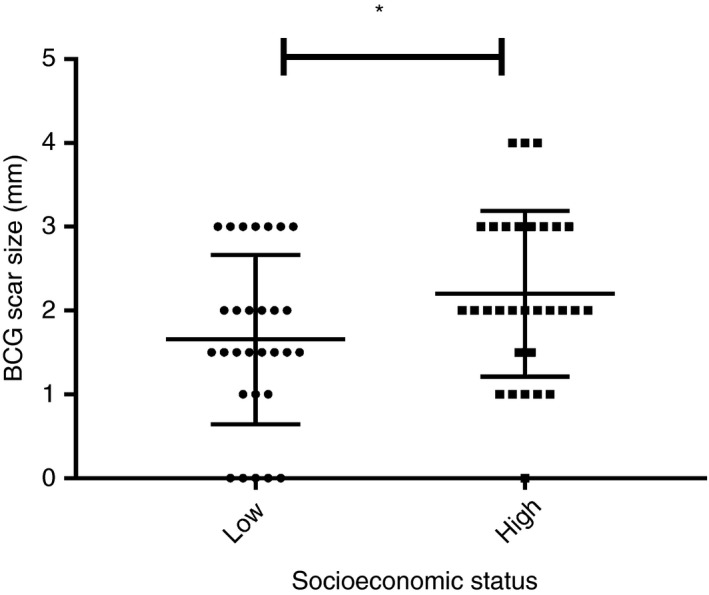
The Bacille Calmette–Guérin scar size in low and high socioeconomic status infants measured at 10 months of age. Data presented as Mean ± SD. * indicates *P* < 0.05 (adjusted for gestational age, sex and weight‐for‐length *z*‐score).

All 59 infants that participated in the follow‐up time points were vaccinated. Among these infants, 89.83% (*n* = 53/59) had developed a BCG scar, while 10.16% (*n* = 6/59; five from low SES and one from high SES) infants did not. Comparison of BCG scar size between the two groups (Figure 2) revealed that infants from high SES have bigger scar size than low SES infants (2.2 ± 0.98 mm *vs*. 1.65 ± 1.01 mm, *P* = 0.041 respectively).

To investigate whether prenatal exposure to infection has an effect on the size of BCG scar, we analysed the association between the size of BCG scar and tIgE, anti‐HAV IgG and anti‐Toxoplasma IgG. We found an inverse relationship between BCG scar size and tIgE (estimates [95% CI] = −0.33 [−0.67 to −0.004] *P* = 0.048) but no association with either anti‐HAV IgG or anti‐Toxoplasma IgG (−0.015 [−0.079 to 0.048], *P* = 0.625; and −0.003 [−0.002 to 0.008], *P* = 0.243 respectively).

**Figure 3:**
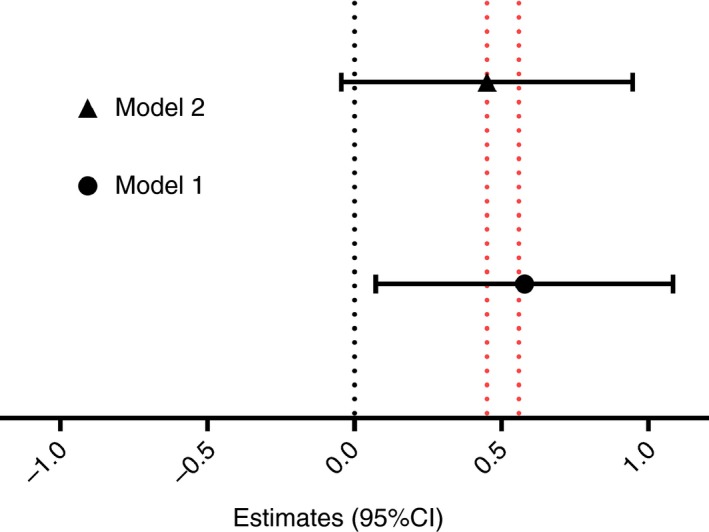
Effects of socioeconomic status on Bacille Calmette–Guérin scar size at 10 months. Model 1: crude (adjusted for gestational age, sex and birth weight‐for‐length *z*‐score); Model 2: Model 1 + cord total IgE. Data presented as beta estimate with 95% confidence interval. [Colour figure can be viewed at http://www.wileyonlinelibrary.com/]

Next, the association between SES and BCG scar size was analysed in multivariate analysis, adjusted by gestational age, sex and Wflz. The results showed that the size of BCG scar remained larger in high SES than low SES infants (estimate [95% CI] = 0.559 [0.083–1.094], *P* = 0.022) (Figure 3 Model 1). Interestingly, when the model was adjusted for tIgE (Figure 3 Model 2), the effect of SES on the size of BCG scar was slightly attenuated (estimates [95% CI] = 0.451 [−0.045 to 0.947], *P* = 0.074) and fell short of significance in terms of predictor of BCG scar size.

### Nutritional status at birth and its association with cord blood adipokines and BCG scar size

**Figure 4:**
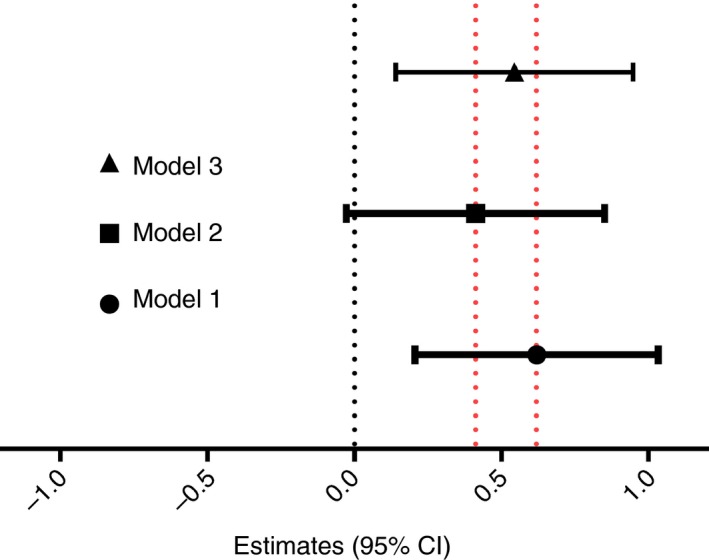
Pathway analysis of nutritional status at birth and Bacille Calmette–Guérin scar size at 10 months. Model 1: Crude (adjusted for gestational age, sex and SES). Model 2: Model 1 + Leptin. Model 3: Model 1 + Adiponectin. Data presented as beta estimate with 95% confidence interval. [Colour figure can be viewed at http://www.wileyonlinelibrary.com/]

To find out whether the size of BCG scar is effected by birth nutritional status through adipokines (Figure 4), we first analysed the association between Wflz and adipokine levels. The results showed that the levels of leptin and adiponectin increased with increasing Wflz (estimates [95% CI] = 1.54 [1.16–2.06], *P* = 0.003 and 1.43 [1.16–1.77] *P* = 0.001 respectively). Analysis of the association between BCG scar size and adipokine levels revealed that the BCG scar size increased with increasing leptin and adiponectin levels (estimates [95% CI] = [9.46 [2.40–37.32], *P* = 0.002]; [13.33 [1.82–97.27], *P* = 0.012] respectively).

Pathway analysis (Figure 4 Model 1) showed that higher Wflz was associated with larger size of BCG scar (estimates [95% CI] = 0.620 [0.206–1.034], *P* = 0.004). Interestingly, when leptin level was also considered (Model 2), the association between nutritional status at birth and BCG scar size is no longer significant and the Wflz effects to BCG scar size was attenuated from 0.620 [0.206–1.034] to 0.412 [−0.027 to 0.852]. However, when adiponectin level was considered (Model 3), this had less effect on the effect of birth Wflz effects on BCG scar size, from 0.620 [0.206–1.034] to 0.545 [0.141–0.949], and Wflz remained as a significant predictor of BCG scar size.

## Discussion

In the present study which involved mothers and their newborns residing in an urban area of Makassar, Indonesia, we found that both SES and nutritional status at birth determine the response towards BCG vaccination measured at 10 months of age. Infants born to low SES families have smaller BCG scar size than infants born to high SES families and tIgE partly contributes to reducing the size of scars. Conversely, infants born with better nutritional status have bigger BCG scars but this association is abrogated by leptin levels at birth.

Similar to our study, a study in school‐aged children in Dominican Republic found positive correlation between BCG scar prevalence and an index of socioeconomic factors 43. In epidemiological studies, low SES has been associated with poorer hygiene practices which in turn could increase exposure to other microorganism or parasites 44. Infections with parasitic helminths are known to induce a strong Th‐2 immune response that can lead to elevated levels of IgE 45, 46. In the current study, we found no statistically significant differences in the prevalence of current helminth infections between mothers with low and high SES; however, the levels of tIgE in mothers/newborns with low SES were significantly higher than mothers/newborns with high SES. This finding indicates that previous helminth infections, or other factors that are not measured in current study such as exposure to allergens 47 including cockroach 48 and dust mites 49, or even tick bites 50, 51 might contribute to the high levels of tIgE. Our previous study in Indonesia, which investigated the development of Th‐2 responses from infancy to 4 years of age reported that children born to mothers with low education or low SES showed stronger development of tIgE responses over time compared to children born to mothers with high SES or high education. In the study, maternal helminth infection status was not the strongest factor determining the Th‐2 polarisation in their children 52. Like infection with *M. tuberculosis*, BCG vaccination induces Th‐1 type immune responses and cause suppression of Th‐2 type responses [53.] Here we found that BCG scar size is inversely associated with the levels of tIgE which is in an agreement with Soysal *et al*. 54 who reported that the presence of scar was associated with lower levels of tIgE.

Nutritional status at birth in our study population was within the normal range and no differences as a function of SES were found. Furthermore, adipokine levels were observed to be positively associated with nutritional status which is consistent with previous studies showed adiponectin and leptin levels increases with increasing body composition at birth 55, 56, 57, 58. The finding that leptin, but not adiponectin, strongly attenuated the relationship between nutritional status and the BCG scar size, suggests that leptin levels and not nutritional status at birth determine the response towards BCG scar formation. To our knowledge, our study is the first to investigate the relationship between leptin levels and BCG scar size. Regarding nutritional status, a recent study in 6‐ to 12‐month‐old babies from Guinea Bissau found that BCG scarification was not associated with nutritional status determined by mid‐upper‐arm‐circumference as well as weight‐for‐age 59.

Leptin is a 16‐kD hormone mainly secreted from adipocytes 60, 61 and has been reported to be positively associated with intrauterine foetal growth 62, birth weight and total body fat content of neonates 63. Leptin is released in the circulation in proportion to the number of adipocytes and acts at the hypothalamus receptor to maintain metabolic homeostasis 61, 64, 65. Besides its role in energy homeostasis, there is also evidence for an immunomodulatory role of leptin. Leptin may shift immune responses towards Th‐1 phenotype 15, 65, 66. In this study, for the first time, we observed that infants with higher neonatal leptin levels had bigger BCG scar size. Although no studies in human have been reported on the role of leptin in vaccine induced protection, in experimental mouse model, Wehrens and colleagues reported that functional leptin receptor signalling is required for mediating an effective protective response against *Helicobacter pylori* 67.

Among infants in this study, 10.16% did not develop BCG scar. We could not find any specific explanation for the absence of scar. The presence or absence of a scar is often used as an indicator of BCG vaccination in a clinical context as well as in health surveys to assess vaccine coverage 68. Our finding was quite similar to that observed by Rani *et al*. 69 and Dhanawade *et al*. 70 where 8.6–9.8% of BCG‐vaccinated infants did not develop any scar. The development of a BCG scar is dependent on several factors such as vaccination technique 59, vaccine strain 59, 71 and cold chain maintenance for a vaccine 72. However, we are not able to pinpoint the reason behind the lack of response to BCG response in our infants.

Some limitations are worth noting, such as the small sample size and considerable loss to follow‐up. However, some interesting data were generated that should be followed up in larger studies powered to discern the role of leptin in modulating BCG scar size.

In conclusion, BCG scar size was influenced by SES and leptin levels at birth. Furthermore, tIgE partly contributed to reducing the size of BCG scar. This study would need to be followed up to determine whether leptin and tIgE affect BCG vaccine efficacy and it also provides a departure point for thinking of strategies whereby leptin levels are increased while tIgE levels are decreased in order to improve responses to BCG vaccine.
